# Prevalence of Gestational Diabetes Mellitus and Its Associated Risk Factors in Boo-Ali Hospital, Tehran

**DOI:** 10.31661/gmj.v9i0.1642

**Published:** 2020-12-26

**Authors:** Mina Etminan-Bakhsh, Sima Tadi, Monireh Hatami, Roksana Darabi

**Affiliations:** ^1^Department of Obstetrics and Gynecology, Faculty of Medicine, Tehran Medical Sciences, Islamic Azad University, Tehran, Iran; ^2^Department of Food science and technology, Faculty of Pharmacy, Tehran Medical Sciences, Islamic Azad University, Tehran, Iran

**Keywords:** Frequency, Gestational Diabetes Mellitus, Risk Factors

## Abstract

**Background:**

Gestational diabetes mellitus (GDM) represents the most common metabolic complication during pregnancy. GDM is associated with maternal and fetal complications. Approximately 7% of all pregnancies are affected by GDM, resulting in more than 200,000 cases worldwide annually, and the prevalence may vary from 1% to 14% among all pregnancies. Accordingly, this study attempted to determine the prevalence and some risk factors of GDM.

**Materials and Methods:**

This hospital-based cross-sectional study was carried out at Boo-Ali hospital in Tehran, the capital of Iran. Four hundred non-diabetics pregnant women with a gestational age of 24-28 weeks who attended the Boo-Ali hospital outpatient department were included in our study. Demographic and anthropometric data including age, gravida, para, gestational age, family history of diabetes, history of GDM, weight, height, and body mass index (BMI) were collected. Blood samples were collected from the women at 24-28 weeks to diagnose GDM by oral glucose tolerance test (OGTT). We measured the 25-OH-D level in participants at 24-28 weeks.

**Results:**

Among the 400 pregnant women, 46 (11.5%) had GDM based on OGTT, and the mean age of GDM women were 30.78± 5.96 years. Among selected variables, BMI ≥25kg/m2, family history, and GDM history were associated with increased risks of GDM (odds ratio=2.49, 95% confidence interval [CI] 1.22–5.07;3.52, 95% CI 1.84–6.70; 19.57, and 95% CI 6.16–62.17, respectively). The association was more robust in the positive GDM history of women.

**Conclusion:**

High prevalence of GDM highlights more attention of health-care givers in screening pregnant women with risk factors. BMI as a modifiable risk factor for GDM needs more attention, and positive family history and previous GDM history should be considered in the core activities of pregnant women.

## Introduction

Gestational diabetes mellitus (GDM) is defined as glucose intolerance of variable degree, with onset or first recognition during pregnancy, whether insulin or only diet modification is used for treatment and whether or not the condition persists after giving birth [1]. GDM represents the most common metabolic complication during pregnancy. GDM is associated with maternal and fetal complications [2]. Placental hormones and increased fat deposits mediate insulin resistance during pregnancy, thereby blocking insulin action to bind its receptors. That is why this condition causes a high level of glucose in pregnancy [3]. Approximately 7% of all pregnancies are complicated by GDM, resulting in more than 200,000 worldwide cases annually, and the prevalence may vary from 1% to 14% of all pregnancies depending on the population studied and the diagnostic tests used [4]. The prevalence of GDM increases with increased levels of obesity and inactivity, which can increase insulin resistance [5], mirroring the increasing rate of type 2 diabetes in the non-pregnant population. The prevalence of gestational diabetes in Iran was estimated at 5.88% in a review study by Almasi *et al*. [6]. Also, Janghorbani *et al*. [7] showed that the prevalence of GDM in Iran varies from 1.3 to 11.9%. The highest and the lowest rate belonged to Orumieh and Ardabil, respectively [7]. In the review study conducted by Sayehmiri *et al*. [8] in 2013, the prevalence of gestational diabetes was 4.9%. The lowest rate belonged to Kermanshah (0.7%), while the highest rate (18.6%) was in Karaj [8]. In 1209 pregnant women who were screened for GDM by Vakili *et al*. in Meibod, the prevalence of gestational diabetes was 27.1% [9]. In a systematic review by Jafari-Shobeiri *et al*., the prevalence of gestational diabetes has been estimated 3.41%, the lowest and the highest prevalence rates were 1.3% and 18.6% in Ardabil and Karaj, respectively [10]. In a review study conducted by Khoshniat *et al*. in 2009 in 11 out of 30 provinces of Iran, a wide range of prevalence for GDM between 1.3%-10% was reported [11]. In studies by Garshasbi *et al*. and Momenzadeh *et al*. in Tehran, the prevalence of GDM was 6.8% and 7%, respectively [12,13]. Several health factors increase the risk of GDM, including older age, previous GDM, high body mass index (BMI >30 kg/m^2^), family history of diabetes, previous macrosomic baby weighing≥ 4.5 kg and ethnicity of high prevalence, mainly South Asian, Black Caribbean, and Middle Eastern [14].

GDM as a common disease during pregnancy, is associated with short- and long-term complications for both mothers and their offspring. GDM increases the chance of diabetes for mothers after giving birth and causes some disorders in both children and mothers during pregnancy, including preterm labor, infection outcomes, polyhydramnios, and an increase in blood pressure. The problems of babies include intrauterine death, inherent disorders, problems related to growth (macrosomia, intrauterine growth retardation), metabolic disorders (hypoglycemia, hypocalcemia), hyperbilirubinemia, polycythemia, birth trauma, respiratory distress syndrome, prenatal mortality, mental retardation, and unjustified death in newborns [15,16]. There was an association between increased blood sugar during pregnancy and childhood obesity at the age of 5-7 years [17], infant macrosomia, and cesarean sections [18,19]. Due to the high heterogeneity among the prevalence of GDM in Iran (%0.7 to %39.4 in Kermanshah and Hamadan, respectively), limited studies in Tehran and its severe complications in mother and fetus, we conducted the present study to identify the prevalence and risk factors of GDM (including vitamin D deficiency) among pregnant women living in Tehran in hopes of a better prevention and screening program which entails early detection to decrease adverse effects and a decrease in patients expenses.

## Materials and Methods

 This hospital-based cross-sectional study was carried out from August 2016 to November 2018 at Boo-Ali hospital in Tehran, the capital of Iran. Pregnant women with a gestational age of 24-28 weeks who attended the outpatient department of Boo-Ali hospital were included in our study. Also, known cases of overt diabetes, detecting GDM before 24 weeks of gestation, patients with a history of vitamin D, and steroid consumption were excluded from the study.

###  Sample Size Calculation

To obtain a sufficient sample size, the highest available prevalence of GDM (27.1%) among Iranian pregnant women [9] was applied in the following sample size formula:

$$n=\frac{{\left(z_{1-\frac{\alpha }{2}}\right)}^{2}(pq)}{d^{2}}$$

Where:

 n=sample size

_Z1-α/2_=z statistic for a level of confidence of 95% (1.96)

 d=determining precision (0.05)

 P=the prevalence of GDM= 27.1%

 q=1-p=72.9%

 An estimated 30% absenteeism or attrition of the respondents was considered. Hence, the sample size needed was 400 pregnant women to avoid any missing data or unwilling participation in the study.

###  Study Participants and Data Collection

 A total of 400 non-diabetic pregnant women with a gestational age of 24-28 weeks participated in our study. Demographic and anthropometric data including age, gravida, para, gestational age, family history of diabetes, history of GDM, weight, height, and body mass index (BMI) were collected. BMI calculated by first trimester or pre-pregnancy weight and gestational age was found out by reliable last menstrual period or first-trimester ultrasound. Blood samples were collected from the women at 24-28 weeks to diagnose GDM by an oral-glucose-tolerance-test (one step OGTT) with 100g glucose. We measured 25(OH)D levels in participants at the same sample at 24-28 weeks. The OGTT should be performed after an overnight fast of at least 8 hours. Based on the International Association of Diabetes and Pregnancy Study Group (IADPSG) criteria, the diagnosis of GDM was made when two of the following plasma glucose values are exceeded: FBS ≥92 mg/dl, 1h≥180 mg/dl, 2h≥155 mg/dl, and 3h≥140 mg/dl. The laboratory kit used to check the serum levels of vitamin D in this study was ADVIA Centaur XP Immunoassay System from Siemens, and based on laboratory information in this method, the level below 20nmol /L was considered to be a vitamin D deficiency.

####  Ethical Considerations

 Informed consent was obtained from all the participants. Ethical clearance was taken from the institutional ethical committee of Tehran Medical Branch of Islamic Azad University (code number: IR.IAU.TMU.REC.1395.187) before starting the study.

###  Data Analysis

 Data analysis was performed using SPSS version 23.0(IBM Corp., Armonk, NY, USA). First of all, data were analyzed descriptively. The mean, median, and standard deviation (SD) for the continuous data and frequency and percentage for the categorical variables were extracted. Exploratory Data Analysis (EDA) was carried out to screen data, identify outliers, describe, and normality distribution of continuous data. The strength of the association of selected variables with GDM odds was evaluated by Odds-ratios (ORs) with 95% confidence intervals (CIs) from cross-tabulation. Independent t-test analysis was calculated to compare the differences means of the selected variables between GDM groups. Also, the chi-square test was used for the testing association between categorical variables. The significant level was considered 0.05.

## Results

 A total of 400 pregnant women participated in our study. The mean age of women was 29± 5.28 years. Most participants were in the age range of 25-34 years (n=250, 62.5%). Among the 400 pregnant women, 46 (11.5%) had GDM based on OGTT, and 354(88.5%) women had normal blood glucose. The mean age of GDM women were 30.78± 5.96 years, and the mean age of non-GDM women were 29.24± 5.17 years. The highest prevalence (18.3%) of GDM was observed in the age group ≥35 years. The mean number of pregnancies in GDM women and non-GDM women was 1.9±0.89 and 1.8±0.83, respectively.

The mean of pre-pregnancy BMI in a total of 400 pregnant women was 26.2± 4.22 kg/m2.^2.^The mean BMI in GDM women and non-GDM women was 28±4.1 and 25.9±4.1 kg/m^2^, respectively. Data analysis showed a significant difference between the means (t [2,398] =3.31, P=0.001). Among the participants, 81 (20.3%) were obese (BMI≥30), and 165 (41.25%) were overweight (BMI 25-29.9). Overall, 246 (61.55%) participants were in the BMI range of ≥25. The prevalence of GDM in normal weight, overweight, and obese women was 6.5%, 11.5 %, and 21%, respectively. Data analysis showed a significant difference between BMI groups and GDM status (χ2 [4,400] =10.96, P=0.004) of which. Among the 33 participants with GDM in current pregnancy and history of previous term or preterm pregnancy, 10 (30%) had GDM in their previous pregnancy. The history of diabetes in the first- and second-degree family was positive in 65% of GDM women and 35% of non-GDM women.

In our study, 243 participants (60.8%) had vitamin D <20nmol/ L. The mean 25(OH)_2_ D_3_ level in GDM women and non-GDM women was 21.98nmol/ L and 19.19nmol/ L, respectively. Among participants with vitamin D deficiency (n=243), 24 women had GDM, and in women with normal vitamin D, 22 women had GDM (Table-1 and 2). The results obtained by odds ratio analysis indicated that GDM was independently associated with BMI, family history and GDM history after controlling for age (Table-3). BMI ≥ 25 kg/m^2^ increased the likelihood of GDM 2.49 (95% CI: 1.22, 5.06). Also, positive family history increased the likelihood of GDM 3.52 (95% CI: 1.84, 6.7), and the likelihood of GDM increased 19.57 times among pregnant women with positive GDM history (95% CI: 6.16, 62.17).

## Discussion

Various prevalence rates have been reported in different studies, but all of them point to an increase in GDM. According to our study, the frequency of GDM was 11.5 % among the study population. However, other studies conducted in Iran include Almasi *et al*. and Vakili *et al*. reported that the frequency of GDM was 5.88% and 27.1%, respectively. The frequency of GDM in Jafari-Shobeiri *et al*. and Sayehmiri *et al*. studies were 3.41% and 4.9%, respectively, and this rate has had changes from 1.3 % to 18.6 % in Jafari-Shobeiri *et al*. study and has had changes from 0.7% to 18.6% in Sayehmiri *et al*. study [6,8,9,10]. The lowest prevalence rate was in Kermanshah, and the highest prevalence rate was in Karaj. In other studies conducted by Garshasbi *et al*. and Momenzadeh *et al*. in Tehran, the prevalence of GDM was 6.8% and 7%, respectively [12,13]. The prevalence of the GDM in the Niyibizi *et al*. study in Rwanda was 8.3 %, in India by Leila *et al*. was 11.8%, and in Yemen by Ali *et al*. and in Peshawar by Bibi *et al*. was 5.1% and 26.3%, respectively [20-23].

Various prevalence rates can arise from the difference in genes, socioeconomic, demography, and culture. The diagnostic method and diagnostic criteria also differed in the mentioned studies. This difference in prevalence is also mentioned in the study done by Schmidtet *et al*. [24]. Furthermore, immigration to Tehran (from different cities and neighboring countries such as Afghanistan) could affect the results. The mean age of our participants was similar or lower to other studies except for the Bibi *et al*. study [23], in which the mean age of participants was higher than the current research. Perhaps the high age of participants was the cause of the high prevalence of GDM in Peshawar [23]. The mean age and BMI of participants in our study were higher compared to Vakili *et al*. study in Meibod and Yazd cities [9]. The mean age and BMI of participants in our study were higher compared to Garshasbi *et al*. [12] study in Tehran. This may be the cause of the higher prevalence in our study. Based on our findings, women aged greater than 34 years were more susceptible to GDM, but this correlation statistically was not significant, unlike previous studies. In Garshasbi *et al*. [12] and Vakili *et al*. [9] studies in Yazd, there was a significant relationship between age and GDM, and age above 25 years was an important risk factor in the study done by Zargar *et al*. [25]. There was no statistically significant association between parity and GDM in our study, which is similar to Momenzadeh *et al*. study [13], but in Vakili *et al*. [9] and Garshabi *et al*. [12] studies, there was a significant relationship between them.

In our study, most of the participants (61.5%) were overweight or obese, and weight gain before pregnancy increases the risk of developing GDM. There were a significant relationship between obesity and GDM in Peshawar, Yemen, Yazd, and many other previous studies, while such a relationship was not found in the study of Niyibizi *et al*. [20].

In our study, the previous history of GDM and the family history of diabetes mellitus were the significant risk factors for GDM in recent pregnancy. A significant association between GDM and previous history of GDM and the family history showed in Garshsbi *et al*. [12], Bibi *et al*. [22], and Ali *et al*. [23] studies. There was a significant relationship between family history of diabetes and GDM, and no significant relationship with GDM in previous pregnancy was observed in the Vakili *et al*. study [9]. The association between vitamin D levels and GDM was still unclear. In this regard, different results have been obtained in studies. Farrant *et al*. [26] in India found no association between second-trimester 25(OH)D levels and GDM. Also, Makgoba *et al*. [27] showed no association between first-trimester 25(OH)D levels and subsequent development of GDM. In a few studies, the association between vitamin D deficiency and GDM has been confirmed, e.g., Maghbooli *et al*. study [28]. Besides, Soheilykhah *et al*. [29] found that the prevalence of GDM was significantly higher in women with vitamin D deficiency. In our study, the prevalence rate of vitamin D deficiency was 61% among the study population. We found that 52% of GDM women and 62% of non-GDM women had 25(OH)D levels <20nmol/ L. According to our findings, vitamin D deficiency was an insignificant variable for GDM. Our results may be affected by a high incidence of vitamin D deficiency in our study population.

## Conclusion

 According to our study, the frequency of GDM was 11.5%, and it seems that its significant risk factors are BMI, previous history of GDM, and family history of diabetes. BMI as a modifiable risk factor for GDM needs more attention; thus, patients should be informed to reach a normal BMI. It is necessary to explain to all pregnant women the importance of screening tests, especially those with risk factors.

## Conflict of Interest

 The authors declared no conflict of interest.

**Table 1:** Frequency of GDM with Selected Risk Factors

**Variables**		**GDM**		**Total**		**P-value**
		**Yes**	**No**	**n**	**%**	
**Age groups, y**	15-24	6	73	79	19.8	0.103
	25-34	27	223	250	62.5	
	≥35	13	58	71	17.8	
**Body mass index, kg/m** ^2^	<25	10	144	154	38.5	0.004
	25-29.9	19	146	165	41.2	
	≥30	17	64	81	20.3	
**Previous history of GDM**	Yes	10	5	15	5.7	<0.001
	No	23	225	248	94.3	
**Family history of diabetes**	Yes	30	123	153	38.2	<0.001
	No	16	231	247	61.8	
**Vitamin D level, nmol/ L**	<20	24	219	243	60.8	0.14
	≥20	22	135	157	39.2	

**Table 2:** Mean Differences of Independent Variables between GDM Groups

**Variables**	**GDM**		**t-value**	**P-value**
	**Yes** **(n=46)**	**No** **(n=354)**		
**Age (year)**	30.83±6.09	29.36±5.13	1.85	0.06
**Body mass index (kg/m** ^2^ **)**	28.41±4.25	25.90±4.2	3.31	0.001
**Fasting blood suger (mg/dl)**	96.20±16.02	81.68±8.21	6.02	<0.001
**1 hour BS (mg/dl)**	201.39±29.23	132.51±26.11	16.60	<0.001
**2 hour BS (mg/dl)**	166.26±30.55	110.92±22.87	14.70	<0.001
**3 hour BS (mg/dl)**	126.63±24.77	88.43±21.87	10.07	<0.001
**Vit. D (25(OH)D), ng/ml**	21.62±15.09	20.19±14.46	1.34	0.18

**Table 3:** Odds-ratios for GDM with Selected Variables

**Variables**		**GDM**		**Odds-ratios** ^†^ ** (95% CI)**	**P-value**
		**Yes** **n (%)**	**No** **n (%)**		
**BMI (n=397), kg/m** ^2^	<25	11 (6.7)	154 (93.3)	2.49 (1.22-5.06)	0.007
	≥25	35 (15.1)	197 (84.9)		
**Age (n=400), y**	≤30	27 (11.7)	203 (88.3)	0.95 (0.51-1.77)	0.50
	>30	19 (11.2)	151 (88.8)		
**Family history (n=400)**	Negative	16 (6.5)	231 (93.5)	3.52 (1.84-6.7)	<0.0001
	Positive	30 (19.6)	123 (80.4)		
**GDM history (n=263)**	Negative	23 (9.3)	225 (90.7)	19.57 (6.16-62.17)	<0.0001
	Positive	10 (66.7)	5 (33.3)		
**Vitamin D level (n=400), ng/ml**	≤20	24 (9.9)	219 (90.1)	1.48 (0.80-2.76)	0.14
	>20	22 (14)	135 (86)		

**†**Unadjusted Odds-ratios **CI**: Confidence interval
